# From Cefepime to Colistin: Managing Multidrug-Resistant Pseudomonas aeruginosa in a Ventilator-Dependent Quadriplegic ICU Patient

**DOI:** 10.7759/cureus.86878

**Published:** 2025-06-27

**Authors:** Esteban Tapias, Marielle Roberts-McDonald, Brian Dunlap, Mikayla Galucia, Muhammad Ali Khalid, Heather L Mateja

**Affiliations:** 1 General Surgery, Western Reserve Health Education, Northeast Ohio Medical University (NEOMED), Warren, USA; 2 General Surgery, Ross University School of Medicine, Bridgetown, BRB; 3 Internal Medicine, Western Reserve Health Education, Northeast Ohio Medical University (NEOMED), Warren, USA; 4 Internal Medicine, Ross University School of Medicine, Bridgetown, BRB

**Keywords:** infectious disease pathology, multidrug-resistant bacteria, pseudomonas aeruginosa, tracheostomy complications, ventilator associated pneumonia

## Abstract

*Pseudomonas aeruginosa* is a Gram-negative bacterium that is commonly associated with nosocomial infections in hospitals. Due to the increasing prevalence of drug resistance, the clinical management and treatment of *P. aeruginosa* are becoming a significant challenge. Here, we report the case of a 21-year-old male with a history of traumatic brain injury, quadriplegia, and ventilator dependence who developed recurrent infections caused by *P. aeruginosa*. Over a span of nine months, the patient was diagnosed with multiple episodes of ventilator-associated pneumonia (VAP) and catheter-associated urinary tract infections (UTIs) requiring intensive care hospitalizations and antimicrobial therapy. Initially, the patient was treated with cefepime and vancomycin; however, due to the rapid rise of multidrug-resistant organisms, the treatment was modified several times with aminoglycosides, meropenem, aerosolized colistin, and ceftolozane/tazobactam. The patient’s case highlights the significant challenge of multidrug-resistant *P. aeruginosa* and underscores the rapid progression of disease secondary to the complexity of treating infections in critically ill patients.

## Introduction

*Pseudomonas aeruginosa* is a Gram-negative, opportunistic pathogen frequently implicated in healthcare-associated infections (HAIs), particularly among critically ill patients. It accounts for approximately 7.1-7.3% of all HAIs, with a disproportionately high burden in intensive care settings [1,2]. Notably, *P. aeruginosa* is responsible for 10-20% of ventilator-associated pneumonias (VAP), approximately 10% of catheter-associated urinary tract infections (CAUTIs), rising to 16% in intensive care units (ICUs), 5.7% of surgical site infections (SSIs), and approximately 4% of central line-associated bloodstream infections (CLABSIs) [1-3]. Its intrinsic resistance mechanisms and ability to acquire additional resistance traits make *P. aeruginosa* a formidable nosocomial pathogen, often challenging to treat and associated with high morbidity and mortality [3].

The primary challenge in treating *P. aeruginosa* infections arises from the bacteria’s ability to resist multiple forms of treatment through a variety of defense mechanisms, including efflux pumps, antibiotic-inactivating enzymes, and the production of biofilm. These mechanisms together have led to the creation of multidrug-resistant (MDR) *P. aeruginosa, *further complicating management and treatment, negatively contributing to increased healthcare morbidity and mortality consequences [1]. Common treatments of *P. aeruginosa* consist of an antipseudomonal beta-lactam (e.g., piperacillin/tazobactam) paired with an aminoglycoside (e.g., amikacin), carbapenems (e.g., imipenem, meropenem), cefepime, and fluoroquinolones also often paired with an aminoglycoside; however, due to rising resistance, these known regimens have become less effective.

In 2017, the World Health Organization classified *P. aeruginosa* as the highest priority, “critical priority,” due to its rapid development of resistance to several commonly used antibiotics [1,4]. MDR *P. aeruginosa* bloodstream infections (BSIs) account for approximately 16.7-28% of all *P. aeruginosa* BSIs, are predominantly hospital-acquired (51% vs. 27% for other Gram-negative BSIs), and are associated with mortality rates as high as 58.8% [1]. One study demonstrated that resistance was notably high for piperacillin/tazobactam (38.5%) and cefepime (32.4%), whereas susceptibility remained relatively higher with amikacin (83.3%), followed by ciprofloxacin at 75.9% [3,5].

To illustrate the clinical complexity and rapid evolution of antimicrobial resistance in *P. aeruginosa* infections, we present the case of a 21-year-old male with quadriplegia and ventilator dependence who experienced multiple episodes of VAP, sepsis, and CAUTIs. Over the course of his illness, the patient developed an MDR strain of *P. aeruginosa*, resulting in treatment challenges compounded by recurrent hospitalizations and opportunistic co-infections.

## Case presentation

**Figure 1:**
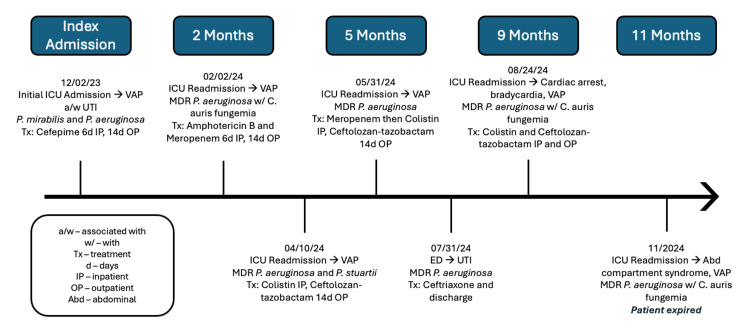
Abbreviated treatment course and development of multidrug-resistant Pseudomonas aeruginosa infection in a 21-year-old male patient despite recommended treatments VAP: ventilator-associated pneumonia; UTI: urinary tract infection; *P. aeruginosa*: *Pseudomonas aeruginosa*; *P. mirabilis*: *Proteus mirabilis*; MDR: multidrug-resistant

We present the case of a 21-year-old male with a complex medical history, including traumatic brain injury, complete C1-C4 quadriplegia, tracheostomy, and ventilator dependence. On December 2, 2023, he was transferred from his nursing rehabilitation facility to the emergency department (ED) with tachycardia and increased mucus production at the tracheostomy site. He was diagnosed with VAP and a concurrent UTI attributed to his indwelling urinary catheter. Upon admission to the ICU, intravenous (IV) vancomycin and cefepime were initiated via a peripherally inserted central catheter (PICC) line. Final cultures identified *Proteus mirabilis* and *P. aeruginosa* in the sputum, while the urine culture showed mixed flora. Vancomycin was discontinued, and cefepime was continued for six days during his ICU stay, then extended for 14 days following discharge. The complete timeline of events for this patient’s treatment course is abbreviated in Figure 1.

Two months later, on February 2, 2024, the patient returned to the ED with diaphoresis and a high-grade fever of 103.6°F. Once again, VAP was suspected, and he was admitted to the ICU. Initial empiric therapy included IV vancomycin and cefepime. Sputum cultures later revealed MDR *P. aeruginosa*, sensitive to gentamicin and tobramycin. Consequently, vancomycin and cefepime were discontinued. Blood cultures grew *Candida auris*, prompting a dual antimicrobial approach with amphotericin B and meropenem. After a six-day ICU stay, the patient was discharged on this regimen for an additional 14 days of outpatient therapy. The patient’s respiratory complications persisted. On April 10, 2024, four months after the initial presentation, he was readmitted from the nursing facility with tachycardia and was again diagnosed with VAP. Sputum cultures identified MDR *P. aeruginosa* and *Providencia stuartii*. Initial empiric antibiotics were replaced by aerosolized colistin based on culture sensitivities. He was subsequently discharged with a 14-day outpatient course of ceftolozane-tazobactam.

Less than two months later, on May 31, 2024, the patient experienced another VAP episode. He was hospitalized and treated initially with meropenem, which was later substituted with aerosolized colistin after six days. Once stabilized, he was again discharged with ceftolozane-tazobactam. By July 31, 2024, the focus had shifted from pulmonary to urinary symptoms. The patient presented with a UTI, and urine cultures grew MDR *P. aeruginosa*. He was discharged from the ED after receiving a single dose of IV ceftriaxone.

On August 24, 2024, nine months after the first episode, he returned to the ED in apparent cardiac arrest. He was stabilized but remained bradycardic, measuring 30 beats per minute. He was admitted to the ICU, where another VAP episode was confirmed. Sputum cultures again showed MDR *P. aeruginosa*, while blood cultures were positive for *C. auris*. He was treated with aerosolized colistin, ceftolozane, and tazobactam, which were continued on an outpatient basis after discharge. However, in November 2024, the patient’s clinical condition deteriorated further. He developed abdominal compartment syndrome in the setting of recurrent *P. aeruginosa *pneumonia and *C. auris* fungemia. Given his poor surgical candidacy and overall prognosis, the family declined operative intervention. The patient subsequently expired.

## Discussion

The rapid development of *P. aeruginosa* antibiotic resistance within a short span and in a young patient underscores the urgent need for timely detection and intervention, as this swift evolution of MDR significantly complicates treatment options and worsens patient outcomes. The case presented above focuses on a 21-year-old male who develops *P. aeruginosa* antibiotic resistance in a span of two months from initial encounter. During the continued treatment with multiple readmissions, antibiotic therapy was decided based on several factors, including recent antibiotic use, recent admission, and ICU placement [3-5].

This patient’s susceptibility to MDR *P. aeruginosa* infection was multifactorial. His status as a young adult with complete C1-C4 quadriplegia, tracheostomy, and ventilator dependence placed him at high risk for HAIs [6]. Prolonged ICU stays, repeated use of broad-spectrum antibiotics, and the presence of indwelling devices (e.g., urinary catheter, tracheostomy) created an ideal environment for nosocomial colonization and infection [6]. His immunocompromised state, impaired mobility, and inability to clear secretions effectively likely contributed to recurrent VAP and complicated infection clearance.

VAP is a complex condition associated with an increased risk of infection, prolonged hospital stays, and a mortality rate exceeding 14% [6]. Therefore, it is crucial to establish an appropriate drug intervention. VAP is initially treated with IV vancomycin and a cephalosporin. Vancomycin, a glycopeptide antibiotic, is the most effective in treating pathogenic bacteria like *Staphylococcus aureus* and *Streptococcus pneumoniae* [6]. One study demonstrated that vancomycin is more effective than cephalosporin, has no cross-resistance with other antibiotics, and few known strains of resistance; therefore, vancomycin combined with cephalosporin is most effective in treating coinfected Gram-positive bacteria and Gram-negative bacteria [6]. Cefepime has a stronger Gram-negative coverage and is more stable against beta-lactamases when compared to the other cephalosporins [7]. This is why it is the drug of choice when concerned about Gram-negative bacteria like *Pseudomonas *or *Proteus*. When looking at this case, the initial treatment was IV vancomycin and cefepime to cover a broad spectrum of bacteria, including *Staphylococcus* and *Pseudomonas*. The initial clinical diagnosis was resolved; however, the patient returned subsequently in two months with new-onset symptoms.

Over a nine-month period, this patient demonstrated an alarming progression from initial *P. aeruginosa* infection to infection with an MDR strain resistant to nearly all first-line therapies. Early isolates were sensitive to cefepime, but subsequent isolates demonstrated resistance to beta-lactams and carbapenems, with sensitivities retained only to aminoglycosides, colistin, and ceftolozane-tazobactam. The rapid emergence of resistance likely resulted from repeated, overlapping exposures to broad-spectrum antimicrobials and prolonged subtherapeutic drug concentrations due to suboptimal dosing or delivery challenges in a critically ill host [2].

Gentamicin, an aminoglycoside, binds to the 16s RNA at the 30s ribosomal subunit in bacterial translation, truncating protein formation [8]. Because of the oxygen-dependent active transport, gentamicin can pass through the membrane of Gram-negative bacteria and additionally release reactive oxidative species, resulting in bacterial cell death [8]. Tobramycin also inhibits the initiation of complex protein translation, promoting antimicrobial killing of Gram-negative bacteria, including *Pseudomonas* [9]. One study proposed the mechanism of tobramycin that has both delayed killing through protein synthesis and immediate killing of the outer bacterial membrane [10]. Across all three studies, the *P. aeruginosa* strains demonstrated consistency of delayed killing requiring a lower intracellular concentration of tobramycin when compared to the immediate killing effect [10]. The study also emphasized aminoglycosides' synergistic combination regimens to maximize bacterial barrier penetration, primarily influenced by tobramycin [10]. Reflecting on the case presented above, antibiotics were switched during the second admission due to sputum cultures positive for MDR *Pseudomonas* and cultures demonstrated sensitivity to gentamicin and tobramycin. Due to the pathophysiology of the antibiotics with an immediate killing effect, the combination of gentamicin and tobramycin was decided. Use of gentamicin and tobramycin was warranted given susceptibility; however, the use of dual aminoglycosides rather than combination with a beta-lactam may have limited efficacy and increased nephrotoxicity risk [5,8,9].

Subsequent shifts to meropenem and aerosolized colistin aligned with escalating resistance, though these agents are often last-line therapies. Ceftolozane-tazobactam was appropriately chosen as a targeted outpatient regimen with preserved activity against resistant strains. However, the recurrence of infection suggests that while susceptibility-guided therapy, biofilm formation, impaired host clearance, or subtherapeutic antibiotic penetration may have led to persistent colonization and clinical failure [10-12]. Meropenem, a carbapenem, is used for broad-spectrum coverage against bacteria, including extended-spectrum beta-lactamase (ESBL), AmpC-producing *Enterobacteriaceae*, and MDR *P. aeruginosa* [11,12]. When considering conditions with *P. aeruginosa* or ESBL-producing bacteria, meropenem is considered first line due to its broad spectrum of activity and retains activity against ESBL and AmpC-producing bacteria [11]. One study focused on patients with VAP, which demonstrated that monotherapy with meropenem responded clinically in 87-91% of patients [11]. Another study focused on VAP patients that demonstrated an inverted U relationship between meropenem monotherapy dose and emerging resistance; co-administration of amikacin reduced the rate of emerging resistance when compared to meropenem monotherapy [12]. Unfortunately, meropenem has developed resistance as well through multiple mechanisms, including porin modification, drug efflux, and beta-lactamase-related hydrolysis [11].

Colistin is one of the first antibiotics with significant activity against Gram-negative bacteria like *P. aeruginosa*, around for the last 50 years [13]. The mechanism of action is not well understood due to the rapidness of its bactericidal effects; however, theorized to be similar to polymyxin B since there is only one amino acid difference [13]. The most common mechanism is modification to lipopolysaccharide at the initial site of action, but colistin is reported to have little resistance secondary to its low usage over the years [13]. One study examined 60 VAP patients who experienced good outcomes with associated aerosolized colistin [14]. This study concluded that the exact role in critically ill patients with MDR Gram-negative bacteria is unclear secondary to a lack of comparative studies examining the different administration routes; therefore, the study reported that aerosolized colistin may be considered an adjunctive therapy in addition to another treatment for VAP patients with MDR Gram-negative bacteria [14].

This case emphasizes several key considerations for managing MDR infections in high-risk patients. First, early consultation with infectious disease specialists and antimicrobial stewardship programs is critical to guide empiric and definitive therapy, minimize unnecessary exposure, and slow resistance development. Second, aggressive source control - in this case, timely consideration of tracheostomy tube exchange or catheter changes - may have helped reduce bacterial load. Third, this case highlights the need to consider combination therapies (e.g., beta-lactam plus aminoglycoside or fosfomycin) and alternative delivery methods (e.g., extended infusion beta-lactams) more aggressively in patients failing standard regimens [2,4-6]. Future care of similar patients should incorporate early de-escalation strategies, routine reassessment of culture data, and earlier transition to agents like ceftolozane-tazobactam when first-line failure is evident. Finally, the presence of *C. auris* coinfection - another MDR organism - should raise concern for hospital-based transmission and may reflect unaddressed infection control issues. Prognostic factors, including nutritional status, frequency of hospital transfers, and persistent colonization, were likely underappreciated and contributed to the patient’s poor outcome.

## Conclusions

This case highlights the significant clinical challenges posed by *P. aeruginosa*, particularly in vulnerable, ventilator-dependent patients with complex comorbidities. Over the course of just a few months, the patient progressed from infection with a susceptible strain to recurrent infections with MDR *P. aeruginosa*, ultimately complicated by co-infection with *C. auris*. Despite appropriate use of culture-guided therapy and escalation to last-line agents, treatment was hindered by rapid resistance development, limited pharmacologic options, and persistent risk factors inherent to the patient's condition. This case underscores the importance of early multidisciplinary intervention, rigorous infection control, and a proactive approach to antimicrobial stewardship. It also emphasizes the need for ongoing innovation in the management of MDR pathogens in critically ill patients, with further research into alternative antimicrobial agents and adjunctive therapies that are critical to improving patient outcomes and preventing the spread of resistant strains.
